# #foodie: Implications of interacting with social media for memory

**DOI:** 10.1186/s41235-020-00216-7

**Published:** 2020-04-16

**Authors:** Jordan Zimmerman, Sarah Brown-Schmidt

**Affiliations:** Department of Psychology and Human Development, Vanderbilt University, 230 Appleton Place, Nashville, TN 37203 USA

**Keywords:** Social media, Memory, Elaborative encoding, Food, Disordered eating

## Abstract

**Background:**

Social media is an increasingly popular outlet for leisure and social interaction. On many social media platforms, the user experience involves commenting on or responding to user-generated content, such as images of cats, food, and people. In two experiments, we examined how the act of commenting on social media images impacts subsequent memory of those images, using Instagram posts as a test case. This project was inspired by recent findings of laboratory studies of conversation which found that describing a picture for a conversational partner boosts recognition memory for those images. Here we aimed to understand how this finding translates to the more ecologically valid realm of social media interactions. A second motivation for the study was the popularity of food- and dieting-related content on Instagram and prior findings that use of Instagram in particular is associated with disordered eating behaviors.

**Results:**

Across two experiments, we observed that commenting on Instagram posts consistently boosted subsequent recognition and that correct recognition increased with comment length. Stable individual differences in recognition memory were observed, and “unhealthy” food images such as chocolates were particularly well remembered; however, these memory findings did not relate to self-reported eating behavior.

**Conclusions:**

Taken together, our findings show that the way in which we engage with social media content shapes subsequent memory of it, raising new questions about how our online lives persist in memory over time, potentially shaping future behavior.

## Significance Statement

Engaging with other persons through social media platforms such as Facebook, LinkedIn, Twitter, Pinterest, and Instagram is becoming increasingly ubiquitous. According to Instagram’s “Year in Review” publicity materials, in 2018, the heart emoji was used over 14 billion times, and the hashtag #metoo was used 1.5 million times (Instagram, 2018). Food-related content is particularly popular on Instagram, with #foodporn tagged on over 206 million posts, #foodie on over 133 million posts, and #vegan on over 83 million posts. A notable feature of Instagram is that users add comments such as “OMG YUMMM,” or hashtag comments such as #food (used over 354 million times) and #fitspo (used over 67 million times). The sheer number of communicative acts demands a better understanding of the cognitive implications of this type of social media engagement. The present research builds on prior studies of conversational interactions which show that describing a picture to a communication partner boosts memory for that image. Here we asked if commenting on Instagram posts similarly boosts memory for those posts. Across two experiments, we observed that commenting on Instagram posts consistently boosted subsequent recognition and that correct recognition increased with comment length. Stable individual differences in recognition memory were observed, and “unhealthy” food images such as chocolates were particularly well remembered, but these memory findings did not relate to self-reported eating behavior. Taken together, our findings show that the way in which we engage with social media images shapes subsequent memory for them, raising new questions about how our online lives persist in memory, potentially shaping future behavior.

## Introduction

Engaging with other persons through language and media is both common and impactful. According to the American Time Use Survey (US Department of Labor, 2019), in 2018, American civilians aged 15 years and older engaged in leisure and sports activities 5.27 h/day, on average, including 38 min/day socializing and communicating and 2.8 h/day watching television. Although the amount of time Americans spent engaging with social media per se is not reported, “playing games and computer use for leisure” was also popular, with an average of 28 min/day, though notably, this varied considerably by age, with 15–19-year-olds reporting 62 min/day and 20–24-year-olds reporting 58 min/day. Blank and Lutz (2016) examined a UK sample and reported that social media platforms such as Facebook, LinkedIn, Twitter, Pinterest, and Instagram are becoming increasingly ubiquitous and that, at the time of their study, Instagram was the fastest-growing of these sites. According to Instagram’s “Year in Review” publicity materials, in 2018, the heart emoji was used over 14 billion times on the platform, and the hashtag #metoo was used 1.5 million times (Instagram, 2018). In its 2017 “Year in Review,” Instagram reported a “global community of 800 million” (Instagram, 2017). One motivation for the present research is the popularity of food-related content on Instagram, such as #foodporn (tagged on over 206 million posts), #vegan (tagged on over 83 million posts), @foodnetwork (9.3 million followers), @foodgod (3.4 million followers), and @feelgoodfoodie (2.1 million followers).

The popularity of social media makes it increasingly important to understand how engaging with this type of media shapes cognition. Previous research suggests that memory for social media content is high among the general population: Memory for Facebook microblogs is significantly higher than memory for sentences from books, news headlines, and even human faces (Mickes et al., 2013). The popularity of health- and dieting-related content on Instagram in particular emphasizes the importance of understanding how the act of viewing and interacting with these images impacts the viewer. Potentially relevant to this question are cognitive processes underlying disordered eating, phenomena that are thought to be complex and multidimensional, being shaped by the external environment and social culture (Culbert, Racine, & Klump, 2015; Levine, Smolak, & Hayden, 1994; Stevenson, Doherty, Barnett, Muldoon, & Trew, 2007). Indeed, some prior work indicates a relationship between the use of media and disordered eating habits (Harrison & Cantor, 1997; Turner & Lefevre, 2017; also see Mejova, Hamed Haddadi, Anastasios Noulas, & Ingmar Weber, 2015). Here, we examined the cognitive processes that occur when engaging with social media content on Instagram.

A notable feature of the Instagram platform is that instead of simply browsing through the content, users can also add comments to the images they see (and see others’ comments as well). These comments often feature evaluations or descriptions of the image, as in “This pasta looks sooo darn good!,” “OMG YUMMM,” or “#foodie.” This act of commenting was the primary focus of the present research. Our research question was inspired by recent findings from the study of conversation that the act of describing an image for another person boosts memory for that image (McKinley, Brown-Schmidt, & Benjamin, 2017; Yoon, Benjamin, & Brown-Schmidt, 2016;). For example, Yoon et al. (2016) examined situations in which pairs of participants viewed four images at a time (on separate computer screens) and took turns describing the images to each other in a task in which the listener had to locate that image and click on it. Despite the fact that the image descriptions were fairly simple (e.g., “the argyle sock,” “the bunny,”) over a series of experiments, speakers consistently exhibited better recognition memory than listeners for these referenced images. Further, memory for viewed but nondescribed images was considerably worse than memory for images that the speaker had described. In a converging finding, McKinley et al. (2017) examined the length of image descriptions and found that longer descriptions promoted better recognition memory for those images.

This observed memory benefit for describing images likely owes to the fact that the labels were generated and produced by the speakers, both of which benefit memory (i.e., the “generation effect” and “production effect”; Fawcett, Quinlan, & Taylor, 2012; Macleod, Gopie, Hourihan, Neary, & Ozubko, 2010; Slamecka & Graf, 1978; Zormpa, Brehm, Hoedemaker, & Meyer, 2019; also see Knutsen & Le Bigot, 2014). Action memory is similarly better for self-performed actions than for observed actions (Koriat, Ben-Zur, & Druch, 1991). These findings can more generally be considered a type of elaborative encoding effect (Bradshaw & Anderson, 1982), where the elaboration of the image with descriptive phrases promotes encoding of that image in memory.

Although it is clear that generating image descriptions for a partner in laboratory tasks with carefully selected images promotes image recognition, an unanswered question is how this might translate into more typical experiences with images in social media contexts. To this end, the present article presents the results of two experiments examining how the act of engaging with social media posts, by commenting on the images, impacts subsequent memory for those posts. Due to the aforementioned prevalence of food- and dieting-related content on this platform and associated comments generated by users (e.g., “We love cucumbers! #instafood #yummy”), we examined several categories of food and non-food-related images and used an established questionnaire to inquire about participants’ eating behaviors. We then modeled the data using a quantitative technique that allowed us to examine whether there were stable individual differences in memory for images in general and in memory for food in particular. If so, we would then be positioned to ask whether a person’s eating behaviors relate to their memory for food-related content on social media. This research question is motivated by previous evidence of selective processing and attentional biases for pictorial stimuli among individuals with eating disorders (Giel et al., 2011; Nikendei et al., 2008; Shafran, Lee, Cooper, Palmer, & Fairburn, 2007; Stormark & Torkildsen, 2004; also see Castellanos et al., 2009), as well as by findings that use of social media platforms by young adults is marginally associated with depressive symptoms (Lup, Trub, & Rosenthal, 2015; cf. Aalbers, McNally, Heeren, Wit, & Fried, 2019). Of note is that platforms such as Instagram are used to share information relevant to eating disorder behavior (see Chancellor, Pater, Clear, Gilbert, & De Choudhury, 2016), and use of Instagram in particular has been associated with higher rates of orthorexia nervosa (an obsession with eating healthy; Turner & Lefevre, 2017). If individuals who experience disordered eating interact with social media on a daily basis and process social media differently on the basis of their symptomology, this could have major implications for how social media platforms affect an already vulnerable population.

## Experiment 1

The aims of Experiment 1 were twofold. First, we tested whether previously observed benefits of generating picture descriptions (McKinley et al., 2017; Yoon et al., 2016; Zormpa et al., 2019) would generalize to the ubiquitous practice of commenting on social media images. Second, we evaluated whether there were stable individual differences in memory for these images, particularly those related to food. If so, this would allow us to take the first step in investigating if and how eating behaviors shape memory for food in social media.

### Methods

This experiment was preregistered with the Open Science Framework Registries (https://osf.io/s5ez8).

#### Participants

Participants were recruited thorough an online platform (Amazon Mechanical Turk; www.mturk.com) and were compensated $4.50 for participating. Criteria for participation were a HIT approval rate of 95% or greater, location as the United States, and number of approved HITS as 100+. Criteria for inclusion in the study were that the participant was a self-reported native English speaker (learned from birth) and that they completed at least 80% of the study. To achieve the final planned sample size of 100 participants, 106 participants completed the study. Six participants were removed for reporting being other than a native speaker of English (*n* = 5), and one was removed for completing the study twice with the same Internet Protocol (IP) address (the second participation in the study was excluded). Thus, the final sample size was 100. The average age of the sample was 37 years (range, 22 to 70). Participants reported gender as female (*n* = 46), male (*n* = 53), or genderqueer (*n* = 1).

#### Materials

The materials were assembled by perusing a large number of Instagram posts gathered from Instagram accounts created by the first author for this purpose. We focused on gathering images from one of five categories. Posts featuring dogs, cats, or nature were used as control images. Posts featuring food served as critical stimuli and were further divided into the categories of “healthy” and “unhealthy” food. Note that categorization of the food images into “healthy” and “unhealthy” was based on group discussion and our intuition about popular cultural beliefs rather than quantitative analysis of nutritional properties. Example “healthy” food images included berries, seafood, and salad. Example “unhealthy” food images included brownies, cheeseburgers, and cake. Posts that originated from private accounts were edited so that the usernames were obscured by overlaying the username with text such as “pics123.” In total, the materials used for the study were 200 Instagram posts, 40 in each of the 5 categories. We intentionally used a 3:2 ratio of control to food-related images in order to obscure the focus on food-related images.

The 200 Instagram posts were rotated across conditions using four lists, counterbalancing which images were shown to participants in the exposure phase (“old” images at test) and which were not shown to participants during exposure (“new” images at test). For old images, we also rotated across lists whether the participant commented on them or not. To create list 1, half of the images in each of the five categories (“healthy food”, “unhealthy food”, cats, dogs, and nature) were randomly selected to be shown to participants in the exposure phase (100 old items); the other half were to be “new” items at test. For each category, half of the “old” images were assigned randomly to the commenting condition, and half were assigned to the no-commenting condition. List 2 then swapped which were commented on and which not. Lists 3 and 4 swapped which were old and new. As a result, on each list, the new items were drawn from among the same categories as the images viewed in the exposure phase. Consequently, there was never a novel item from a completely new category that was not previously encoded. For example, across the whole experiment (study and test), participants saw five total images of pizza, some of which were new items and some of which were old (which ones were old vs. new depended on the list). Please see the Appendix for a list of all of the food stimuli in the study for list 1. Each participant was randomly assigned to complete the trials on a single list (list 1, *n* = 27; list 2, *n* = 29; list 3, *n* = 23; list 4, *n* = 21).

#### Procedure

##### Exposure phase

After consenting to participate, participants were given the following instructions: “In the following section, you will see a series of Instagram posts. Some will require you to generate an original comment, while others will not. Please think of appropriate and thoughtful comments for the pictures you will see, and simply attend to the posts that do not require comments. Treat the following experience as you would scrolling through your own social media feed.” The instructions did not mention that there would be a subsequent memory test. Participants then clicked to the next screen and began viewing a series of 100 Instagram posts, one per page. The posts were presented in a different random order for each participant. For half of the posts, the participants were prompted to provide a comment using the same phrasing as on the Instagram platform (i.e., “Add a comment….”). For comment trials, there was no restriction on the length of the comment, though the participant needed to type something in order to proceed to the next trial. These trials were self-paced.

##### Test phase

After viewing the 100 Instagram posts, participants were then asked to complete a series of 17 math problems. This task took about 5 min and was intended to bring memory performance off ceiling because recognition memory performance for pictures can be high (Shepard, 1967); this is a method used in our prior work with success (Yoon et al., 2016). Participants were then told, “In the following section, your memory will be tested on the images you were previously shown. If an image is presented that you have previously seen (one you saw in the first part of the study), select ‘OLD.’ If an image is presented that is new and not previously shown, select ‘NEW.’” They viewed a series of 200 images, half of which were old and seen in the exposure phase (20 images from each of the 5 categories), and half of which were new (20 new images from each of the 5 categories). The images were shown one at a time in a different random order for each participant, and the participant was asked to respond “old” or “new” for each image.

#### Additional measures

Following the recognition memory task, participants completed the Eating Disorder Examination Questionnaire (EDE-Q). The EDE-Q is the self-report version of the Eating Disorder Questionnaire, which is used to determine the frequency and severity of behavioral features and the psychopathology of eating disorders. The questionnaire provides a global score of severity as well as four subscale scores that correspond to certain aspects of psychopathology. The four subscales are restraint, eating concern, shape concern, and weight concern. The range of ratings for each item is 0 to 6, with 6 being the most frequent or severe. To obtain a subscale score, the average of the ratings for the relevant items is calculated. To obtain a global score, the sum of the four subscale scores is divided by 4 (Fairburn, Cooper, & O’Connor, 2014). This examination is reported to be a valid measure of eating disorder symptomology (Mond, Hay, Rodgers, Owen, & Beumont, 2004), and higher values on each of the scales reflect higher symptomology. Finally, participants were asked to report their age, gender, and ability in the English language.

##### Predictions

Recent studies in our laboratory revealed that following conversation, partners had better memory for pictures that they described themselves than for ones their partner described (McKinley et al., 2017; Yoon et al., 2016). If this generation benefit extends to written comments in online communication, we hypothesized that memory would be better for Instagram posts for which individuals generated original comments than for posts that were passively viewed. Further, we expected that the likelihood of correct recognition would increase the longer the comment. This pattern of findings would be expected if posting comments on social media promoted elaborative encoding of the post (Bradshaw & Anderson, 1982), compared with simply viewing them.

There is, however, good reason to think that commenting may not benefit picture memory. Social media images are often selected to be glossy and captivating, and recognition memory for pictures tends to be good (Shepard, 1967). Thus, ceiling effects may obtain, such that commenting has no effect on memory despite the use of a filled delay between study and test. Another possibility is that an elaborative encoding benefit of commenting trades off with an attentional shift toward the “Add a comment…” box where the comment is typed, and inward toward one’s own reflections on the image. Memory for item and context can trade off (Gopie & MacLeod, 2009; Jurica & Shimamura, 1999; Koriat et al., 1991), such that manipulations that benefit item memory impair or have no effect on context memory. If so, commenting may boost memory for the comment, but not the picture; in linguistic terms, it would be the reference and not the referent that receives the boost. Such a pattern of findings would circumscribe the scope of generation-based memorial benefits following conversation, pointing to a distinction between reference and referent.

In addition to effects of commenting, we tested the hypothesis that individual differences in memory for food vs. nonfood could be predicted by self-reported disordered eating behaviors. Support for the idea that memory for food might be linked with disordered eating comes from findings of attentional processing differences for food-related stimuli in persons with an eating disorder (Giel et al., 2011; Nikendei et al., 2008; Shafran et al., 2007; Stormark & Torkildsen, 2004) and a link between hunger/satiation and attentional processing of food-related stimuli (Mogg, Bradley, Hyare, & Sui, 1997; Placanica, Faunce, & Job, 2002; Stockburger, Schmälzle, Flaisch, Bublatzky, & Schupp, 2009). These findings, along with evidence of a relationship between the use of media and disordered eating habits (Harrison & Cantor, 1997; Turner & Lefevre, 2017; also see Mejova et al., 2015), led to the predictions (1) that there would be stable individual differences in memory for food (vs. nonfood) images and (2) that these individual differences would be related to a measure of disordered eating behavior.

Critically, however, examining the relationship between this digital generation effect and behaviors associated with disordered eating first requires us to demonstrate reliable individual differences in memory for the images and for food-related images in particular. To preview, while persons did exhibit stable individual differences in recognition memory, the difference in memory between food and nonfood items was itself not a stable property of the individuals we tested. Unfortunately, this prevented us from testing hypotheses relating memory for food-related Instagram posts to disordered eating behaviors.

### Results

**Fig. 1:**
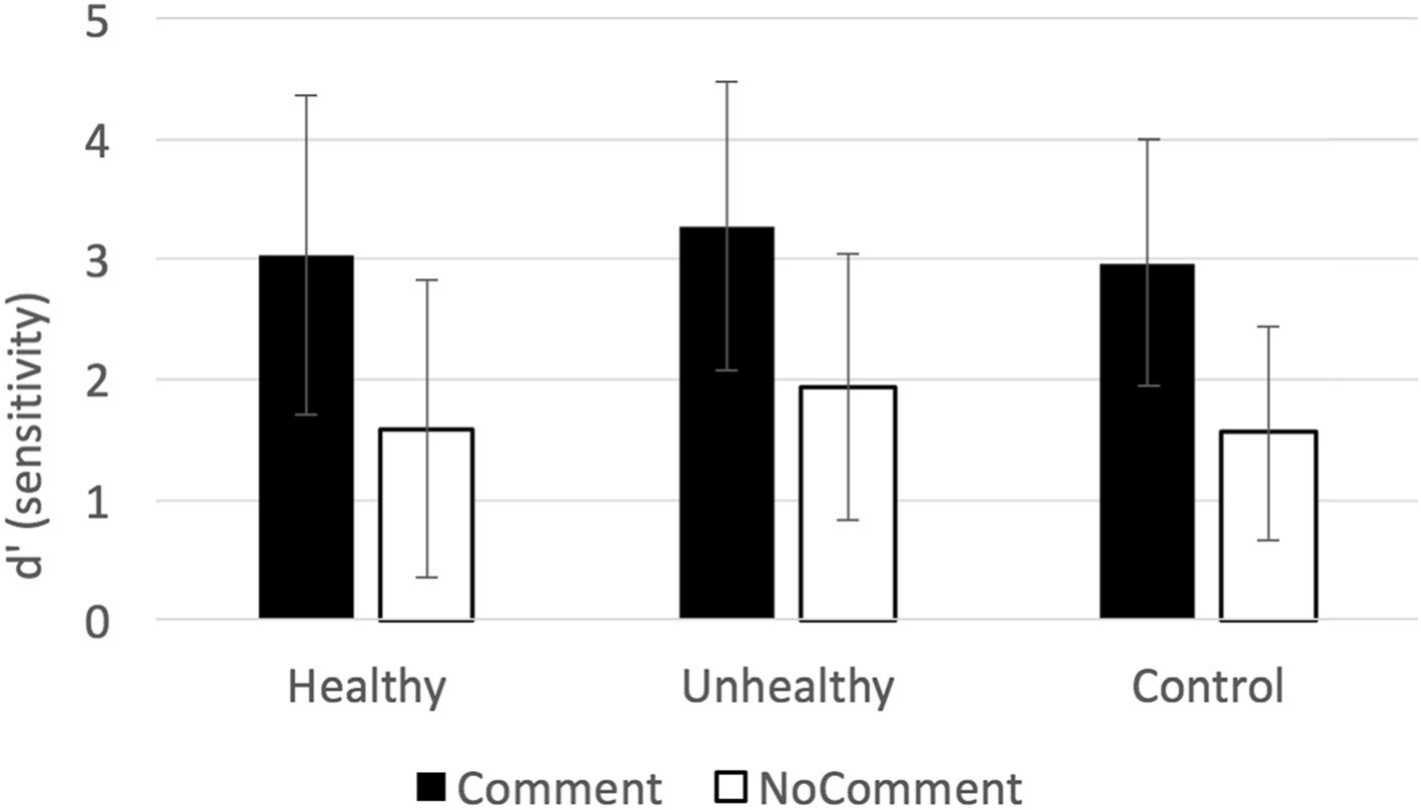
Experiment 1: Illustration of memory sensitivity (*d*′) by condition. Error bars indicate by-participant standard deviation

Each participant commented on 50 of 100 viewed Instagram posts, followed by an old–new recognition memory test. Performance in the recognition memory task is illustrated using a measure of memory sensitivity (*d*′) in Fig. 1.

The primary analyses focus on memory for the posts, analyzed using a logistic mixed-effects approach to a signal detection theoretic analysis (see Fraundorf, Benjamin, & Watson, 2013; Wright, Horry, & Skagerberg, 2009). In addition, we characterize the relationship between the form of the comments and memory and finally explore whether there are stable individual differences in these effects.

#### Memory for Instagram posts

A logit-link mixed effects model for memory judgments (old = 1; new = 0) was fit to the 19,999 recognition memory judgments in this dataset; note that one data point was lost due to computer error. Planned analyses of fixed effects include orthogonal Helmert codes for item type (old vs. new) and whether the image had been commented on (comment vs. no comment). We also consider two different effects of image type using a dummy-coding scheme where the control images (nature, dogs, or cats) were dummy-coded as baseline, allowing us to test if memory for healthy food differed from baseline and separately whether memory for unhealthy food differed from baseline. Note that the effects of item type and commenting can be interpreted as simple effects at the reference level (control images), and interactions with item type test whether these effects differ for healthy and for unhealthy food.

The model included participants and items as crossed random effects. An initial model that included only the random intercepts revealed that there was very little variance in the intercepts by item, though slightly more variability among participants. On the basis of lack of item variability and the lack of hypotheses regarding individual item differences, we did not attempt models with random slopes by item. While our analysis plan was to relate a person-level covariate (EDE-Q score) to the memory data, attempts to include random slopes by persons for each of the fixed effects were met with convergence failures. Using a backward-fitting approach (Barr, Levy, Scheepers, & Tily, 2013), we simplified the model until we identified a model that included random slopes by person for the effects of item type (old vs. new), and commenting (for old items, whether the participant commented on the image). While this model failed to converge, refitting the model with different optimizers using the allFit function (Bates et al., 2018) revealed consistent estimates for each of the fixed effects to two decimal places, except for the intercept and the (nonsignificant) interaction between commenting and healthy food, which were consistent across optimizers only to one decimal place. For the random effects, estimates for the by-person random intercept and slopes were consistent to two decimal places, except for the by-participant random slope for item type (old vs. new) and commenting, which were consistent only to one decimal place. This model was taken to be satisfactory and is presented in Table 1.

**Table 1:** Experiment 1: Memory by condition, model with random slopes^a^

**Fixed effects**	**Estimate**	**SE**	**z Value**	***p*value**
(Intercept)	0.086	0.076	1.128	.259
**Commenting** (commented items = 0.5, noncommented items = −0.5, new = 0)	**2.613**	**0.144**	**18.090**	**<.0001**
**Item type (old vs. new)** (commented = 0.5, noncommented = 0.5, new = −1)	**2.786**	**0.126**	**22.114**	**<.0001**
Commenting * Healthy food	−0.122	0.146	−0.836	.403
Commenting * Unhealthy food	**−0.335**	**0.151**	**−2.218**	**<.05**
Item type * Healthy food	**−0.218**	**0.076**	**−2.869**	**<.01**
Item type * Unhealthy food	**0.217**	**0.081**	**2.692**	**<.01**
**Random effects**	**Variance**	**SD**	**Correlations**	
Item (intercept)	0.206	0.454		
Subject (intercept)	0.389	0.623		
Item type (old vs. new)	1.343	1.159	0.27	
Commenting	1.285	1.134	−0.12	.47

^a^Number of observations: 19,999, 200 items, 100 participants

The intercept term in the model was not significant, indicating there was no evidence for a significant response bias (participants responded “old” and “new” at similar rates). A significant effect of item type (*z* = 22.11, *p* < .0001) indicated good memory for the previously viewed images. A significant effect of commenting indicated that for previously viewed images, they were more likely to be correctly recognized if the participant had commented on them (*z* = 18.09, *p* < .0001). These effects were qualified by interactions with item type. Healthy food was remembered less well than control images (z = − 2.87, *p* < .01), whereas unhealthy food was remembered better than control images (*z* = 2.69, *p* < .01). In addition, the effect of commenting was smaller for unhealthy food images than for control images (*z* = − 2.22, *p* < .05), possibly due to the fact that memory was overall better for the unhealthy food images.

#### Effect of comment length on memory for Instagram posts

This exploratory analysis investigated whether the length of the Instagram comments modulated memory for those images. This analysis was restricted to old items for which participants generated a comment, as the predictor variables are defined for those items only. The number of words per comment ranged from 1 to 45 (median = 3, mean = 4.18). On average, comments were longer for pictures that would ultimately be correctly recognized (mean number of words = 4.30, SD = 3.24) than for pictures that were not recognized (mean = 2.86, SD = 2.13). A logit-link mixed effects model for memory judgments (old = 1; new = 0) was fit to the data. Fixed effects include a centered measure of the number of words used to comment on the picture. The same dummy-coding scheme as in the primary analysis was used, where control images (nature, dogs, or cats) were dummy-coded as baseline, allowing us to test if the effect of comment length was different for healthy food and for unhealthy food (compared with baseline). Note that the effects of the number of words can be interpreted as simple effects at the reference level (control images) and interactions with item type test whether these effects differ for healthy food and for unhealthy food.

Participants and items were included as random intercepts. A null model was initially fit to the data and indicated very little variability by items but some variability by participants. Thus, the effect of commenting was included as a by-participant random slope. This model converged. Models with more complex random effects structures failed to converge with warnings indicating singular fits. Thus, results of this converged model were taken to be satisfactory and interpreted (Table 2). The significant intercept term indicates that for these commented-on images (all of which were old), correct recognitions were more likely than not (*z* = 18.94, *p* < .0001). A significant effect of word count (*z* = 6.09, *p* < .0001) shows us that for each additional word produced in the comment, the odds ratio of correct recognition was 1.37 times greater at test. A significant interaction between word count and healthy images (*z* = − 2.43, *p* < .05) indicated that the effect of commenting was smaller for healthy food images than for control images (dogs, cats, or nature).

**Table 2:** Experiment 1: Effect of comment length on correct identification of old images

**Fixed effects**	**Estimate**	**SE**	***z* Value**	***p*Value**
**(Intercept)**	**2.969**	**0.157**	**18.936**	**< .0001**
**Word count**	**0.313**	**0.051**	**6.085**	**< .0001**
Words*Healthy	**−0.137**	**0.057**	**−2.425**	**<.05**
Words*Unhealthy	−0.090	0.059	−1.530	0.126
**Random effects**	**Variance**	**SD**	**Correlations**	
Item (intercept)	0.318	0.564		
Participant (intercept)	1.206	1.098		
Word count	0.026	0.162	0.290	

#### Participant variability and Individual differences

In order to ask questions about individual differences in these processes, it is first necessary to determine whether the models provided evidence that there were stable (reliable) individual differences in our memory measures. To this end, for the analysis of recognition memory presented in Table 1, we extracted the by-person random effects as well as the standard errors of those random effects using the “arm” package in R (Gelman, Su, Yajima, Hill, et al., 2018). Following Cho, Shen, and Naveiras (2019), we calculated the model-based reliability of the item type (old vs. new) and comment effects. Rho can be interpreted as the ratio of the estimated variance theta over the observed variance theta, with values closer to 1 indicating better reliability. Rho for the item type effect was fairly high, .882, indicating stable individual differences in memory for the pictures. Rho for the effect of commenting was less reliable, .654.

As noted above, more complex models including random slopes for image type (healthy and unhealthy food) failed to converge, and refitting these models revealed inconsistent fits with different optimizers as well as singularities (random effects parameters being at or near zero), indicating poor model fit. Together, these findings indicate that there was some consistent variability by persons in their memory for the images and in the effect of commenting on those pictures. However, we were not able to extract a stable measure of individual differences in memory for healthy or unhealthy food in particular (above and beyond overall measures of memory), making it impossible to conduct planned analyses of the relationship between memory for food and the EDE-Q measure of eating behavior.

For the second analysis of the relationship between comment length and memory (Table 2), inspection of the random effects indicated that while there was some variability in the by-person intercept (reflecting the correct recognition rate) the effect of word count on correct recognition varied little across participants. Model-based estimates of reliability (Cho et al., 2019) indicated a mediocre rho value for the by-person intercept (.557); the extremely small variance for the random slope for word count resulted in a negative rho (− 2.842). These findings indicate that there was very little evidence for reliable individual differences in these effects, and they were not explored further.

The person-level covariates in this sample include participant age, and the EDE-Q measures (restraint, eating concern, shape concern, weight concern, and the global score). Descriptive statistics for these variables are shown in Table 3.

**Table 3:** Person-level covariates in Experiments 1 and 2, with means and standard deviations by participants in parentheses^a^

	Experiment 1	Experiment 2
N	100	150
Age	36.77 (10.59)	26.17 (3.31)
Education	–	3.13 (1.40)
Restraint	1.38 (1.6)	1.84 (1.85)
Eating concern	0.65 (0.95)	1.03 (1.19)
Shape concern	1.78 (1.66)	2.59 (1.88)
Weight concern	1.65 (1.52)	2.37 (1.74)
Global score	1.39 (1.33)	1.96 (1.51)

^a^Note that the education measure was not included in Experiment 1. The Eating Disorder Examination Questionnaire measures are calculated for 143 participants in Experiment 2. See text for details

We took an exploratory approach to examining bivariate correlations between the EDE-Q measures (restraint, eating concern, shape concern, weight concern, and the global score), participant age, and the memory measures (by-person random effects for response bias, overall memory, and the effect of commenting). These correlations were computed and interpreted with respect to a Bonferroni-adjusted alpha level of .0013. The bivariate correlations revealed the expected relationships among the EDE-Q subscales and the overall global score (Table 7 in Appendix). In addition, the by-person random effects for memory and commenting were positively related (*r =* .487, *p* < .0001), indicating that participants with better memory also tended to benefit more from commenting on the images. There was also an unexpected positive correlation between the memory random effect and age (*r =* .356, *p* < .001). The lack of stable individual differences in memory for healthy and unhealthy food in particular prevents examination of a relationship between those food types and scores on the EDE-Q.

### Discussion

The results of Experiment 1 revealed, for the first time, that the process of commenting on social media images boosts memory for those images. This effect can be interpreted as a type of generation effect (Slamecka & Graf, 1978). Further, the fact that longer comments produced better recognition extends prior findings from studies of in-laboratory image descriptions (McKinley et al., 2017) to a class of images – Instagram posts – that are both ubiquitous and socially relevant. This effect can be considered a type of elaborative encoding effect (Bradshaw & Anderson, 1982) such that the more elaborately the participant commented on the post, the better the memory. The memorial benefit for commented-on pictures may be enhanced in part by a longer time spent on trials where participants generated a comment. (Unfortunately, the study software did not provide information about timing.) If so, such a timing difference would reflect properties of the natural phenomenon we intended to study: commenting on social media images. Here we have shown that the memorial boost conferred by generating comments in communication extends to a new type of item with a high degree of social relevance: social media.

## Experiment 2

The primary aim of Experiment 2 was to replicate the findings of Experiment 1. Second, we aimed to examine whether interacting with Instagram posts, in particular posts about food, would be particularly memorable for a sample of participants who are at a higher risk for exhibiting disordered eating behaviors. While we did not find consistent individual differences in food-related Instagram posts in Experiment 1, the participant sample may have been too broad to capture the population of interest. Lifetime prevalence of eating disorders in US adults is higher in women than in men and in younger adults (Hudson, Hiripi, Pope Jr, & Kessler, 2007; Udo & Grilo, 2018; also see Cheng, Perko, Fuller-Marashi, Gau, & Stice, 2019). Experiment 2 is therefore a replication of Experiment 1, but with a participant sample restricted to young females.

### Methods

The project was preregistered with the Open Science Framework (https://osf.io/dqrge). Experiment 2 was identical to Experiment 1, with two exceptions. First, we restricted the sample to participants who identified as female and who were between the ages of 18 and 30. Second, we asked participants additional questions about their education level.

#### Participants

As planned, we present an analysis of data of 150 participants. We used a 50% larger sample in this study because whereas the results of Experiment 1 showed a large generation effect, attempts to fit models with more complex random effects structures were met with convergence issues. Thus, the larger sample size was selected with the aim of reducing convergence issues.

We used Amazon Mechanical Turk Premium qualifications to select a sample of female participants, aged 18–30. As in Experiment 1, criteria for participation is a HIT approval rate of 95% or greater, location as the United States, and number of approved HITS as 100+. Criteria for inclusion in the study were that the participant was a self-reported native English speaker (learned from birth) and that they completed at least 80% of the study. Repeated HITS from the same IP address (including IP addresses from Experiment 1) are excluded (the initial HIT is included).

Five additional participants completed at least 80% of the study but were excluded due to missing demographic information (*n* = 3), reporting gender as male (*n* = 2), or reporting as a non-native English speaker (*n* = 1). We also note that despite using Premium qualifications to select participants who were between the ages of 18 and 30, of the 150 participants included in the analysis, 17 participants reported an age of 31 or 32. Inclusion of these 17 participants in the final sample results in an average age of 26.2 years (SD = 3.3) versus 25.5 if they are excluded. As this was unanticipated and the central tendency does not shift much by including them, we chose to include these 17 participants in the final sample rather than remove them in a *post hoc* decision. The majority of participants (89%) had completed at least some college.

#### Materials

The materials were identical to Experiment 1, except that we additionally asked about participants’ educational experience. Specifically, at the end of the study, participants were asked the highest level of school they had completed or the highest degree received, the year they graduated from college, the year they received an undergraduate or bachelor’s degree, and whether they were currently enrolled in school, along with the level of current enrollment. These questions were included in order to better characterize the sample, as prior work aims to understand the social and physical factors that contribute to unhealthy eating behaviors among college students (LaCaille, Dauner, Krambeer, & Pedersen, 2011).

##### Predictions

We expected to replicate the finding that recognition memory for commented-on posts would be better than posts that were passively viewed and that the likelihood of correct recognition would increase with longer comments. If participants were to demonstrate reliable individual differences in memory for food-related images in particular, this would allow us to then test the hypothesis that higher rates of disordered eating behaviors would result in better memory for food-related images. To preview, however, we again found that participants exhibited stable individual differences in recognition memory but that differences in memory between food and nonfood items was itself not a reliable measure.

### Results

**Fig. 2:**
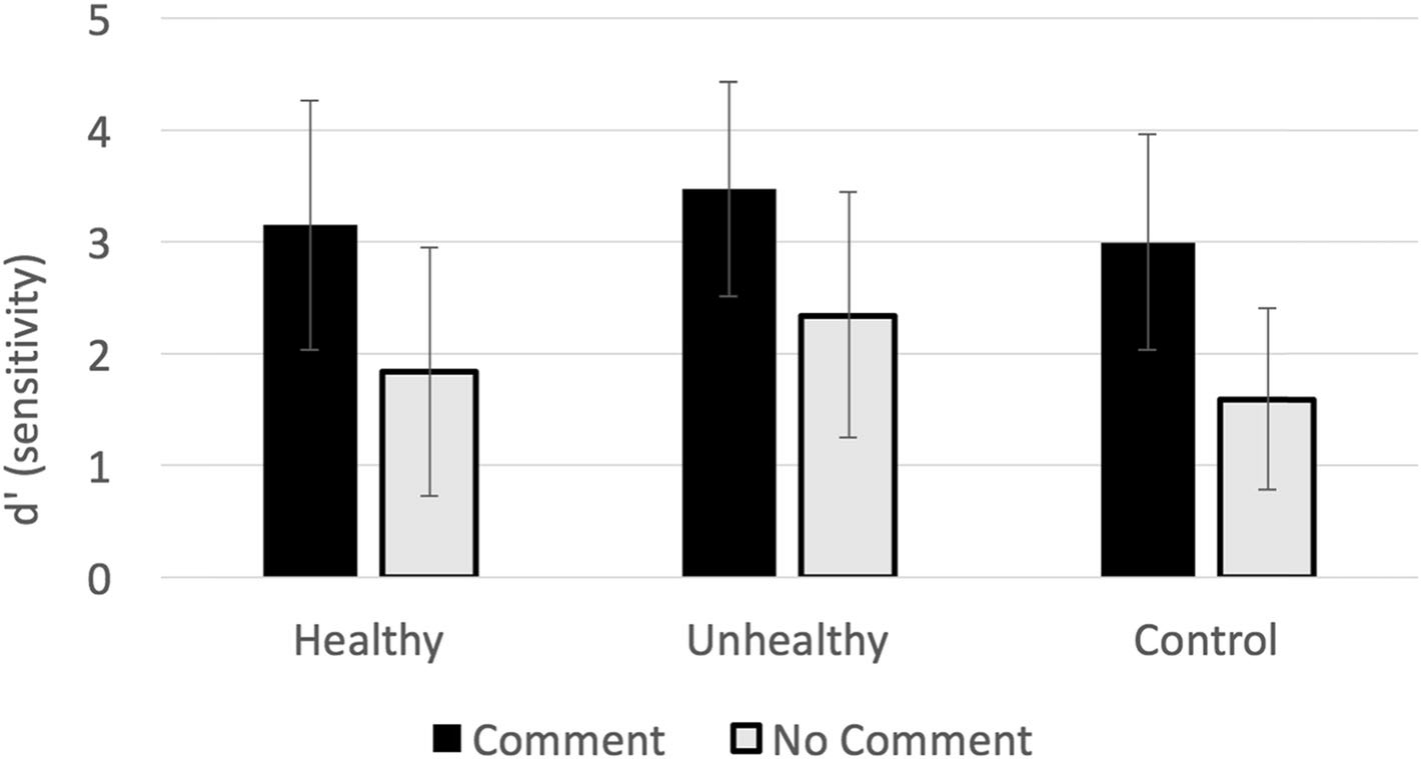
Experiment 2: Illustration of memory sensitivity (*d*′) by condition. Error bars indicate by-participant standard deviation

As in Experiment 1, our primary analyses focused on memory for the posts, analyzed using a logistic mixed-effects model. In addition, we characterized the relationship between the form of the comments and memory, and finally we explored whether there were stable individual differences in these effects. For illustration purposes, the memory data are plotted using a measure of memory sensitivity (*d*′) in Fig. 2.

A logit-link mixed effects model for memory judgments (old = 1; new = 0) was fit to the 29,999 recognition memory judgments; note that one data point was lost due to computer error. Planned fixed effects include orthogonal Helmert codes for item type (old vs. new) and whether the image had been commented on (comment vs. no comment). Effects of image type were coded using the same dummy-coding scheme as before, comparing memory for nonfood (baseline) with memory for healthy and unhealthy food. As before, the effects of item type and commenting can be interpreted as simple effects at the reference level (control images), and interactions with item type test whether these effects differ for healthy and for unhealthy food. As in Experiment 1, a null model indicated very little by-item variability but some by-subject variability. Attempts to include the full random slopes structure by person were met with convergence failures. A backward-stepping procedure was used to remove random slopes one by one to improve model fit. The final model resulted in warnings, but refitting the model using the allFit function indicated that estimates for the fixed effects were identical to two decimal places for each of the optimizers, indicating satisfactory model fit for inferences regarding the fixed effects. This model (Table 4) included random intercepts for subjects and items and a random slope for the item type effect by subjects.

**Table 4:** Experiment 2: Number of observations: 29,999, 200 items, 150 participants

**Fixed effects**	**Estimate**	**SE**	***z*Value**	***p*Value**
(Intercept)	0.127	0.067	1.894	.058
**Commenting** (commented = .5, noncommented = −.5, new = 0)	**2.419**	**0.065**	**37.408**	**<.0001**
**Item type** (commented = .5, noncommented = .5, new = − 1)	**2.736**	**0.091**	**30.225**	**<.0001**
Commenting * Healthy food	−0.211	0.125	−1.684	.092
Commenting * Unhealthy food	**−0.433**	**0.135**	**−3.218**	**<.01**
Item type * Healthy food	0.062	0.064	0.961	.336
Item type * Unhealthy food	**0.625**	**0.071**	**8.806**	**<.0001**
**Random effects**	**Variance**	**SD**	**Correlation**	
Item	0.1949	0.4415		
Subject	0.4497	0.6706		
Item type (subject)	1.0073	1.0036	0.09	

A significant effect of item type (*z* = 30.23, *p* < .0001) indicated good memory for the previously viewed images. A significant effect of commenting indicated that previously viewed images were more likely to be correctly recognized if the participant had commented on them (*z* = 37.41, *p* < .0001). These effects were qualified by interactions with image type. Unhealthy food was remembered better than control images (*z* = 8.81, *p* < .0001). As in Experiment 1, the effect of commenting was smaller for unhealthy food images, compared with control images (*z* = − 3.22, *p* < .05); this interaction may be due to the overall better memory for unhealthy food in the first place.

#### Effect of comment length on memory for Instagram posts

Given the observed positive relationship between comment length and recognition in Experiment 1, this planned analysis was expected to reveal a positive relationship between comment length and successful recognition. As before, the analysis was restricted to old items for which participants generated a comment. The number of words in the comments ranged from 1 to 35 (mean = 4.12, median = 3). On average, comments were longer for pictures that would ultimately be correctly recognized (mean number of words = 4.15, SD = 1.90) compared with pictures that were not recognized (mean = 3.40, SD = 2.32). A logit-link mixed effects model for memory judgments (old = 1; new = 0) was fit to the data. Fixed effects included a centered measure of the number of words used to comment on the picture. The same dummy-coding scheme as in the primary analysis was used, comparing control images with healthy and unhealthy food. Note that the effect of the number of words can be interpreted as a simple effect at the reference level (control images) and interactions with item type test whether this effect differs for healthy and for unhealthy food images.

Participants and items were included as random intercepts. A null model was initially fit to the data and indicated very little variability by items but some variability by participants. Thus, the effect of comment length was included as a random by-participant slope. This model converged. Attempts to fit more complex models with interactions with image type were met with convergence warnings indicating singular fits; thus, the results of this model were taken to be satisfactory and interpreted (Table 5). The significant intercept term indicates that for these commented-on images (all of which were old), correct recognitions were more likely than not (*z* = 23.34, *p* < .0001). A significant effect of word count (*z* = 4.61, *p* < .0001) shows that for each additional word produced in the comment, the odds of correct recognition were 1.20 times greater. The remaining fixed effects were not significant, indicating similar memory performance across the image types.

**Table 5:** Experiment 2: Effect of comment length on recognition of old images

**Fixed Effects**	**Estimate**	**SE**	***z*Value**	***p*Value**
**(Intercept)**	**3.119**	**0.134**	**23.344**	**< .0001**
**Word count**	**0.182**	**0.039**	**4.612**	**< .0001**
Words*Healthy	0.033	0.050	0.662	.508
Words*Unhealthy	−0.002	0.050	−0.048	.962
**Random Effects**	**Variance**	**SD**	**Correlation**	
Item (intercept)	0.560565	0.74871		
Participant (intercept)	1.212417	1.1011		
Word count	0.008459	0.09197	0.43	

#### Participant variability and individual differences

The final model of recognition memory in Experiment 2 included a random slope for the item type effect by persons (Table 4). As indicated above, models that included more complex random slopes failed to converge, indicating that there was not strong support for consistent individual differences in memory for healthy and unhealthy food over nonfood images. We calculated the model-based reliability for the by-participant effect of item type (old vs. new). As in Experiment 1, Rho for the item type effect was fairly high, .863, indicating stable individual differences in memory for the pictures.

For the model of the effect of comment length on correct recognition (Table 5), inspection of the random effects parameters indicated that while there was some variability in the by-person intercept (reflecting individual differences in correct recognition), the effect of word count on correct recognition varied little across participants. Model-based estimates of reliability (Cho et al., 2019) indicated a poor rho value for the by-person intercept (.572); the extremely small variance for the random slope for word count resulted in a negative rho value (− 4.23). These findings indicated that there was very little evidence for reliable individual differences in these effects; thus, we do not explore them further.

Measures of individual differences in this sample include the same EDE-Q measure as in Experiment 1, as well as age and education level. EDE-Q scores for seven participants were missing, and those participants were excluded from this analysis. Descriptive statistics for the remaining 143 participants are shown in Table 3. The EDE-Q scores were higher in this sample than for the participants in Experiment 2, consistent with prior work indicating a higher incidence of disordered eating in women (Cheng et al., 2019; Hudson et al., 2007). Education level was recoded as a numeric variable ranging from 0 to 6 to reflect highest level of educational achievement attained (0 = less than high school degree; 6 = post-graduate degree).

Exploratory bivariate correlations between the EDE-Q measure, participant age, education level, and the by-person random effects for response bias and overall memory were computed and interpreted with respect to a Bonferroni-adjusted alpha level of .0013. The bivariate correlations revealed the expected relationships among the EDE-Q subscales and the overall global score (Table 8 in Appendix). None of the other relationships were significant. As in Experiment 1, the lack of stable individual differences in memory for healthy and unhealthy food in particular prevents examination of a relationship between those food types and scores on the EDE.

### Discussion

Experiment 2 repeated Experiment 1 on a sample of young adults who reported their gender as female. Consistent with the literature on disordered eating behaviors, the average EDE-Q scores for this group were higher, though still within 1 SD of published norms (Fairburn et al., 2014). Despite these differences, the central results of Experiment 1 were replicated. We replicated the finding that the process of commenting on social media images boosts memory for those images, and we replicated the finding that longer comments result in more correct recognitions. We also replicated the curious effect that unhealthy images were correctly recognized more often than control images and that, in turn, the effect of commenting was smaller for unhealthy food images. While the experiment failed to provide strong evidence for systematic individual differences in memory for food-related Instagram posts, thus preventing our relating food-specific memory to EDE-Q scores, we replicated the finding that there were stable differences in overall memory for the pictures. This finding indicates that future work could use this paradigm as a starting point to build an explanatory model of individual differences in memory for social media images.

## General discussion

Our most robust finding is that the act of commenting on an Instagram post boosts memory for that post and, further, that the odds of correct recognition increase the longer the comment. This finding, which was consistent across five image categories (“healthy” and “unhealthy” food, cats, dogs, and nature) indicates that the way in which a user engages with content on social media shapes memory for it. The Instagram platform is designed to allow users to engage with content by commenting on it (with emojis, text, and other in-app actions such as “liking” or sharing). Our findings indicate that engaging in this way promotes the ability to later recognize those images. Another notable feature of the Instagram experience is that users select what types of accounts to follow. While we did not explore the implications of choosing what content to view, the fact that the observed relationship between commenting and memory was apparent for all image types indicates that whatever content the user chooses to follow, when they choose to engage with that content, it is likely to impact memory.

We investigated memory for images of food in particular due to their popularity on Instagram (e.g., #foodporn, #foodie). Prior arguments that individuals with disordered eating process food-related stimuli differently from healthy participants (Nikendei et al., 2008; Shafran et al., 2007), along with the fact that platforms such as Instagram are commonly used to share information relevant to disordered eating behavior (see Chancellor et al., 2016), make it important to understand how interacting with these types of stimuli impacts the user. The present findings provide clear evidence against the idea that there are stable individual differences in memory for food, at least in a nonclinical sample. Instead of an individual trait, the observed memory benefit for “unhealthy” food over control images may be a more general phenomenon, potentially related to the fact that food, particularly high-calorie food, is rewarding (see Frank et al., 2010; Simmons, Martin, & Barsalou, 2005).

While we do not find consistent individual differences in memory for food in particular, it is important to consider the fact that some users may curate their content in order to view primarily food- and/or dieting-related content. Our findings show that engaging with social media images through commenting extends the user experience beyond the in-the-moment experience of the platform, promoting subsequent ability to recognize that content later. Thus, an important question for future work is whether the observed memory effects impact future real-world behaviors associated with that content, particularly in clinical samples. Social media users will retain memory for whatever content they choose to view; thus, choices over what to view may be relevant to considerations of how social media use impacts the user. Finally, the lack of reliable individual differences in memory for food, despite a consistent finding across experiments of better memory for unhealthy food vs. control, may owe to the fact that low between-participant variance may be necessary to produce a stable experimental effect – the “reliability paradox” (Hedge, Powell, & Sumner, 2018).

We also found that the “unhealthy” food images, which included posts featuring items such as cake, cheeseburgers, and pizza, were remembered better in both experiments than our control images, which included cats, dogs, and nature pictures. This unexpected finding may relate to the attention-captivating properties of high-calorie food (Castellanos et al., 2009), along with arguments that thinking about the survival relevance of a stimulus boosts memory for it (Nairne & Pandeirada, 2010). A limitation of this explanation, however, is that Nairne and Pandeirada (2010) found that it is processing an item’s relevance to survival that boosts memory, regardless of whether those items were in fact relevant to survival. Of course, explaining this item-specific effect would require further study and ruling out other explanations related to specific item properties.

This project was inspired by previously observed benefits of generating picture descriptions for subsequent recognition memory (McKinley et al., 2017; Yoon et al., 2016; Zormpa et al., 2019). Here we show that this result extends to the socially relevant domain of social media images. Consistent with the present results are findings that sharing personal memories on social media platforms improves memory for memories that were shared compared with those that were not shared (Wang, Lee, & Hou, 2016; see Stone & Wang, 2019, for discussion). One issue that Stone and Wang (2019) raised is that information that persons choose to share may be inherently more memorable. We note that in the present research, participants did not choose what to comment on; yet, we similarly observed a benefit to memory for engaging with the images.

However, other research indicates that engaging with media and technology more generally can impair memory. For example, the use of media to record or share thoughts during an experience harms subsequent memory for that experience compared with not using media to memorialize the experience (Tamir, Templeton, Ward, & Zaki, 2018). Similarly, the act of photographing objects can harm memory for those objects (Henkel, 2014). The presence of smartphones nearby in the room impairs performance on working memory tests (Ward, Duke, Gneezy, & Bos, 2017), suggesting that even the potential to disengage may harm one’s ability to fully process the current experience. Further, frequent use of social media is associated with poorer academic outcomes (Feng, Wong, Wong, & Hossain, 2019) and with memory failures (Sharifian & Zahodne, 2019).

The present finding that engaging with social media through commenting improved memory for it is not necessarily inconsistent with this evidence of technology-related memory impairment. The difference in findings may relate to the fact that extracting the self from an experience long enough to memorialize it with media detracts from the experience itself. By contrast, generating a comment about a social media image may enhance the experience of the image through elaborative encoding (Bradshaw & Anderson, 1982). Commenting on social media images may also invite rehearsal (Roediger and Karpicke, 2006) or offloading (Storm & Stone, 2015) effects, promoting recollection (for discussion, see Marsh & Rajaram, 2019; Stone & Wang, 2019). Thus, the impact of engaging with technology on memory may depend on the user experience and whether the experience interferes with the processing of the to-be-encoded event in the first place. More generally, understanding the cognitive implications of engaging with the Internet and social media may require a better understanding of how usage patterns change with time and with increased use (see Storm, Stone, & Benjamin, 2017).

### Conclusion

The tremendous popularity of social media as an outlet for leisure and social interaction makes it increasingly important to understand how engaging with social media shapes cognitive processes. Consider the fact that on Instagram alone, the comment #foodporn has been used over 206 million times, #food over 354 million times, and even #omgyum over 38,000 times. The results of two experiments show that generating comments such as these changes the way those images are memorialized, offering an ecologically valid replication and extension of prior work (McKinley et al., 2017; Yoon et al., 2016). The fact that “unhealthy” food images such as chocolates were particularly well remembered raises new questions about the impact of engaging with food-related content on subsequent cognition. Taken together, our findings show that the way in which we engage with social media content shapes subsequent memory for it, raising tantalizing questions about how our online lives persist in memory over time, potentially shaping future behavior.

## Data Availability

The raw, de-identified data associated with this article are available at (https://osf.io/8gafu/). Materials are available upon request.

## Appendix

**Table 6:** Descriptions of the “healthy” and “unhealthy” food items for List 1 in the study^a^

**Healthy food (old)**	**Unhealthy food (old)**
1. Diced watermelon with lemon	1. Chocolate filled donuts
2. Grilled shrimp + broccoli	2. Pepperoni pizza, stuffed crust
3. Salad with avocado and brown rice	3. Slice of Oreo cheesecake
4. Strawberry yogurt + whole strawberries	4. Cinnamon rolls
5. Grain bowl with tofu + broccoli	5. Whole chocolate cake filled with candy
6. Sliced fruit with fruit juice	6. Chocolate- and Oreo-covered strawberries
7. Toast with smoked salmon and zucchini	7. Fudge brownies with pretzels
8. Corn, avocado, and tomato salad	8. Slice of red velvet cake
9. Acai bowl topped with berries and banana	9. Peanut butter brownies with M&Ms
10. Salmon, veggies, and quinoa	10. Bacon double cheeseburger
11. Sautéed vegetables	11. Cheese and jalapeño pizza
12. Tomato, cucumber, and avocado salad	12. Pecan praline French toast
13. Spaghetti squash	13. Chicken wings + ranch dressing
14. Tropical smoothie bowl	14. Chocolate cannolis
15. Bowl of berries and kiwi	15. Milkshakes with whipped cream
16. Vegetable kabobs	16. Ice cream cookie sandwich
17. Vegetable sandwich	17. Whole Nutella cake
18. Bowl of strawberries, raspberries, and mangos	18. Cheesy pizza missing a slice
19. Fruit and nut granola	19. Oreo candy bar
20. Sautéed shrimp + orange slices	20. Six whole chocolate cakes
**Healthy (new)**	**Unhealthy (new)**
1. Bowl of watermelon, kiwi, and orange	1. Slice of chocolate cake
2. Rice bowl with tofu, greens, and tomato	2. Cheesy pizza
3. Fruit and nut cereal with milk	3. Whole chocolate layer cake
4. Tropical fruit bowl + flowers	4. Whole chocolate truffle cake with cream
5. Grilled shrimp + asparagus	5. Chocolate-drizzled brownies
6. Breakfast porridge with banana and mango	6. Bacon cheeseburger with fries
7. Fruit + nut yogurt bowl	7. Oreo cookie milkshakes
8. Bowl of strawberries, raspberries, and watermelon	8. Box of chocolate-covered donuts
9. Pumpkin + spinach pasta serving	9. Cookie dough + sprinkles on ice cream
10. Vegan dish of chicken, veggies, and brown rice	10. Box of stuffed bagel bites
11. Fruit kabobs	11. Cookies and cream brownies
12. Cauliflower rice, cucumber, and salmon bowl	12. Basil pizza
13. Bowl of mangos, kiwi, blueberries, and dragon fruit	13. Multiple Oreo candy bars
14. Grain bowl with veggies, greens, and salmon	14. Cheese pizza on the beach
15. Banana and almond butter granola	15. Large jalapeño and pepperoni pizza in a box
16. Blueberries	16. Barbecue sandwich with fries
17. Avocado + egg Cobb salad	17. Chocolate and Kit Kat candy bar whole cake
18. Caprese avocado toast	18. Fried chicken + fries and dip
19. Smoothie bowl with strawberries, cocoa nibs, and bananas	19. Donut topped with candy, sprinkles, and cookie dough
20. Cinnamon and raisin oatmeal	20. Cadbury Creme Egg stuffed brownie

^a^Across the four lists, we counterbalanced which items were old vs. new

**Table 7:** Experiment 1: bivariate correlations between person-level covariates of age and Eating Disorder Examination Questionnaire subscales and global scale, as well as correlations with model-derived by-person estimates for response bias, memory, and the effect of commenting^a^

	Bias	Memory	Comment	Restraint	Eating	Shape	Weight	EDE global	Age
Bias	1.000								
Memory	0.288	1.000							
Comment	−0.226	**0.487**	1.000						
Restraint	0.088	0.034	0.043	1.000					
Eating	0.275	−0.126	− 0.255	**0.579**	1.000				
Shape	0.236	0.004	− 0.093	**0.675**	**0.812**	1.000			
Weight	0.197	−0.016	− 0.103	**0.644**	**0.795**	**0.958**	1.000		
EDE global	0.229	0.021	−0.097	**0.821**	**0.828**	**0.953**	**0.939**	1.000	
Age	0.055	**0.356**	0.309	0.042	−0.052	0.055	0.014	0.038	1.000

Correlations that are significant at a Bonferroni-corrected alpha level of .0013 (for 36 comparisons) are marked in boldface. The Eating Disorder Examination Questionnaire (EDE) included the following scales: Restraint, Eating, Shape, Weight, EDE global

**Table 8:** Experiment 2: bivariate correlations between person-level covariates of age, education, and Eating Disorder Examination Questionnaire subscales and global scale, as well as correlations with model-derived by-person estimates for response bias and memory^a^

	Bias	Memory	Education	Restraint	Eating concern	Shape concern	Weight concern	Global	Age
Bias	1								
Memory	0.122	1							
Education	0.048	0.046	1						
Restraint	0.010	0.006	−0.097	1					
Eating concern	−0.072	0.028	−0.129	**0.664**	1				
Shape concern	−0.034	0.034	−0.095	**0.689**	**0.783**	1			
Weight concern	−0.073	0.008	−0.077	**0.690**	**0.771**	**0.946**	1		
Global	−0.043	0.020	−0.107	**0.851**	**0.867**	**0.950**	**0.946**	1	
Age	0.087	−0.037	0.183	0.082	−0.086	0.021	0.007	0.016	1

Correlations that are significant at a Bonferroni-corrected alpha level of .0013 (for 36 comparisons) are marked in boldface. The Eating Disorder Examination Questionnaire (EDE) included the following scales: Restraint, Eating, Shape, Weight, EDE global

## Abbreviation

EDE-Q: Eating Disorder Examination Questionnaire
